# Alteration of mRNA 5-Methylcytosine Modification in Neurons After OGD/R and Potential Roles in Cell Stress Response and Apoptosis

**DOI:** 10.3389/fgene.2021.633681

**Published:** 2021-02-03

**Authors:** Huan Jian, Chi Zhang, ZhangYang Qi, Xueying Li, Yongfu Lou, Yi Kang, Weimin Deng, Yigang Lv, Chaoyu Wang, Wei Wang, Shenghui Shang, Mengfan Hou, Hengxing Zhou, Shiqing Feng

**Affiliations:** ^1^Department of Orthopaedics, Tianjin Medical University General Hospital, Tianjin, China; ^2^International Science and Technology Cooperation Base of Spinal Cord Injury, Tianjin Key Laboratory of Spine and Spinal Cord, Tianjin Medical University General Hospital, Tianjin, China; ^3^Department of Orthopaedics, Qilu Hospital, Cheeloo College of Medicine, Shandong University, Jinan, China; ^4^Shandong University Center for Orthopaedics, Cheeloo College of Medicine, Shandong University, Jinan, China; ^5^Key Laboratory of Immuno Microenvironment and Disease of the Educational Ministry of China, Department of Immunology, Tianjin Medical University, Tianjin, China

**Keywords:** m^5^C methylation, epitranscriptome, ischemia-reperfusion injury, oxygen-glucose deprivation/reoxygenation, cell stress, apoptosis

## Abstract

Epigenetic modifications play an important role in central nervous system disorders. As a widespread posttranscriptional RNA modification, the role of the m^5^C modification in cerebral ischemia-reperfusion injury (IRI) remains poorly defined. Here, we successfully constructed a neuronal oxygen-glucose deprivation/reoxygenation (OGD/R) model and obtained an overview of the transcriptome-wide m^5^C profiles using RNA-BS-seq. We discovered that the distribution of neuronal m^5^C modifications was highly conserved, significantly enriched in CG-rich regions and concentrated in the mRNA translation initiation regions. After OGD/R, modification level of m^5^C increased, whereas the number of methylated mRNA genes decreased. The amount of overlap of m^5^C sites with the binding sites of most RNA-binding proteins increased significantly, except for that of the RBM3-binding protein. Moreover, hypermethylated genes in neurons were significantly enriched in pathological processes, and the hub hypermethylated genes RPL8 and RPS9 identified by the protein-protein interaction network were significantly related to cerebral injury. Furthermore, the upregulated transcripts with hypermethylated modification were enriched in the processes involved in response to stress and regulation of apoptosis, and these processes were not identified in hypomethylated transcripts. In final, we verified that OGD/R induced neuronal apoptosis *in vitro* using TUNEL and western blot assays. Our study identified novel m^5^C mRNAs associated with ischemia-reperfusion in neurons, providing valuable perspectives for future studies on the role of the RNA methylation in cerebral IRI.

## Introduction

Posttranscriptional RNA modifications have important biological significance in RNA metabolism. The most widely studied RNA posttranscriptional modification is N6-methyladenosine (m^6^A). Moreover, “writers,” “erasers,” and “readers” of the m^6^A modification have been identified. Increasing evidence shows that m^6^A exhibits a wide range of effects on mRNA metabolism, including mRNA stability, translation, and splicing (Cao et al., 2016; Meyer and Jaffrey, 2017; Zhao et al., 2017). N1-methyladenosine (m^1^A), 5-methylcytosine (m^5^C), pseudouridine (Ψ), and other modification types also play an important role in the posttranscriptional regulation of genes (Carlile et al., 2014; Li et al., 2015; Dominissini et al., 2016; Amort et al., 2017; Yang et al., 2017). Cytosine methylation is a new type of RNA modification first identified in tRNA and rRNA (Helm, 2006; Schaefer et al., 2009). Using advanced high-throughput techniques combined with next-generation sequencing (NGS), m^5^C modification sites were also found in many mRNAs. The main regulatory proteins that were identified include the NOP2/Sun RNA Methyltransferase Family Member 2 (NSUN2, known as an m^5^C “writer”) and Aly/REF export factor (ALYREF, known as an m^5^C “reader”; Yang et al., 2017). Existing studies have demonstrated that the m^5^C methylation modification of mRNA can regulate key biological processes, such as mRNA nuclear effects, maintenance of mRNA stability, and neural stem cell differentiation (Flores et al., 2017; Yang et al., 2017; Chen et al., 2019b).

Ischemia-reperfusion injury (IRI) could cause extensive tissue and organ damage, and the core mechanism of IRI is blood vessel remodeling after deprivation of the blood supply to a certain area or organ and blood reperfusion after tissue and organ ischemia (Eltzschig and Eckle, 2011). Current studies have demonstrated that ischemia and reperfusion can cause tissue and organ damage through a variety of pathophysiological mechanisms. The main pathological mechanisms include activation of the complement system, calcium overload, reduction in oxidative phosphorylation, endothelial dysfunction, activation of the apoptotic signaling pathways, and an increase in the free radical concentration (Eltzschig and Eckle, 2011**;** Duehrkop and Rieben, 2014). In brain tissue, ischemia and hypoxia are closely related to many neurological diseases, such as traumatic brain injury (TBI), acute ischemic stroke, and iatrogenic cardiopulmonary bypass surgery (Wiberg et al., 2016; Lopez et al., 2017; Zhao et al., 2018).

In addition, epigenetic effects play an important role in nerve damage caused by IRI. Studies have demonstrated that miR-424 can enhance the levels of DNA methyltransferase 1 (DNMT1) and histone 3 lysine 27 trimethylation through the NFIA/DNMT1 signaling pathway, thereby preventing astrogliosis after cerebral IRI in mice (Zhao et al., 2019). Simultaneously, after cerebral IRI, the m^6^A RNA demethylase AlkB homolog 5 (ALKBH5) selectively demethylates Bcl2 transcripts, thereby preventing the degradation of Bcl2 transcripts and enhancing Bcl2 protein expression (Xu K et al., 2020). Therefore, epigenetic modifications of nerve tissue may play an important role in the protection of nerve function and microenvironment improvement after injury. However, under cerebral IRI conditions, the landscape and potential functions of m^5^C modifications of mRNA remain unclear.

Thus, in this study, we aimed to gain a deeper understanding of the m^5^C methylation of neurons after IRI. To this end, we established an oxygen-glucose deprivation/reoxygenation (OGD/R) model with primary cerebral neurons to simulate cerebral IRI *in vivo*. Sequencing of bisulfite-treated RNAs (RNA-BS-seq) was performed to analyze whether the methylation modification of mRNA was significantly altered after OGD/R treatment. The results showed that m^5^C methylation modifications were abundant in neurons. Simultaneously, after OGD/R induction, numerous novel m^5^C sites were identified in neuronal mRNAs that were associated with various disease-related pathways. In addition, we examined the potential connections among the m^5^C methylation sites, protein-binding sites and chromosome distribution. In general, our research provided a comprehensive description of the epigenetic mechanism of m^5^C modification in neurons after OGD/R and afforded basic information for further research on the function and specific mechanism of m^5^C after IRI in neural tissues.

## Materials and Methods

### Primary Cerebral Neuron Isolation and Culture

Murine primary cortical neurons were isolated from embryos of pregnant C57/BL6 mice as previously described (Hilgenberg and Smith, 2007; Sciarretta and Minichiello, 2010). Briefly, the cortex of the embryonic mice was dissected in high-glucose DMEM (Gibco, Cat# 31053028) at 4°C. Then, the cortical tissue was cut into 1-mm^3^ pieces and centrifuged at 800 rpm for 5 min at room temperature. The tissue was incubated with papain solution (10 U/ml; Worthington, Cat# LS 03126) for 15 min at 37°C in a 5% CO_2_ incubator and dissociated into single cells by gentle trituration. Then, single cells were resuspended in DMEM-HG containing 10% fetal bovine serum (Gibco, Cat# 10099-141) and 1% penicillin/streptomycin (Invitrogen, Cat# 15140148). Finally, neurons were plated on poly-l-lysine (Sigma-Aldrich, Cat# P4832)-precoated cell culture dishes at 1.0 × 10^6^ cells/ml. After 4 h, the previous medium was replaced with neurobasal medium (Gibco, Cat# 21103049) containing 2 mmol/L glutamine (Gibco, Cat# 25030081), 1% B-27^TM^ Supplement (50X; Gibco, Cat# 17504044), and 1% P/S (Invitrogen, Cat# 15140148). The neuronal media were changed every 3 days.

### Oxygen–Glucose Deprivation/Reoxygenation

Neuronal medium was removed, and neurons were washed with phosphate-buffered saline (PBS; Sigma-Aldrich. Cat# D8537) supplemented with 1% P/S several times. To initiate OGD, neuron cells were cultured in glucose-free DMEM (Gibco, Cat# 31053028). Then, neurons were placed in hypoxic conditions (37°C, 94% N_2_, 21% O_2_, and 5% CO_2_) for 3 h. Then, the glucose-free DMEM was changed to normal neuronal culture medium, and neurons were incubated under normal incubatory conditions (37°C, 5% CO_2_) for 24 h. The culture conditions were simulated for reperfusion. Neurons treated without OGD served as a control group.

### RNA Extraction

TRIzol reagent (Invitrogen Corporation, CA, United States) was used to extract RNA from the primary cultured neurons following the manufacturer’s protocol, and the NEBNext^®^ rRNA Depletion Kit (New England Biolabs) was used to reduce the rRNA content. The RNA concentration of each sample was measured by NanoDrop ND-1000 (Thermo Fisher Scientific). The ratio of OD260/OD280 value was used to assess the purity of the RNA index. When the OD260/OD280 value ranges from 1.8 to 2.1, RNA is considered pure. Electrophoresis was performed on a denaturing agarose gel to evaluate RNA integrity.

### RNA-BS-Seq and Identification of m^5^C Sites

Briefly, RNA was bisulfite-converted and purified using the EZ RNA Methylation Kit (Zymo Research). Then, the TruSeq Stranded Total RNA Library Prep Kit (Illumina) was used to construct the RNA libraries. The library quality was assessed by a BioAnalyzer 2100 system (Agilent Technologies, Inc.). Library sequencing was performed on an Illumina HiSeq instrument with 150-bp paired-end reads. Paired-end reads were quality controlled by Q30, and low-quality reads were removed after 3′ adaptor trimming by Cutadapt software (v1.9.3). STAR software was used to match clean reads of the input library to the reference genome (version mm10; Dobin et al., 2013), which were obtained from the UCSC database, and meRanGh software (a component of meRanTK) was used to align the clean reads of the bisulfite-treated library to the reference genome (Rieder et al., 2016). meRanCall was used to extract each methylated cytosine (C) site in the genome, and meRanCompare was used for the identification of differentially methylated sites. Finally, the m^5^C sites were considered credible with an m^5^C methylation level ≥ 0.1 and a coverage depth ≥ 10.

### Analysis of the m^5^C Distribution

According to the provided method, the distribution map of the m^5^C locus on the chromosome was drawn according to the m^5^C locus information (Hao et al., 2020). The m^5^C locus information was annotated using BEDTools (Quinlan and Hall, 2010), and m^5^C sites in the mRNA were mapped to five regions: 5′UTR, start codon, CDS, stop codon and 3′UTR. In addition, 21-nt sequences proximal to the m^5^C locus on both sides were extracted with BEDTools, and logo plots were generated using WebLogo. The distribution of methylation peaks was plotted using MetaPlotR software (Olarerin-George and Jaffrey, 2017). Profiles of the RNA-binding protein (RBP) binding sites were downloaded from POSTAR (Hu et al., 2017), and the available binding sites were combined into one BED file and used to analyze the overlap with the m^5^C sites.

### Bioinformatics Analysis

To explore the function of the m^5^C modification after OGD/R, the m^5^C sites were selected according to the criteria of the m^5^C methylation level ≥ 0.1 and coverage depth ≥ 10 in all three repetitions. As a comprehensive and excellent biometric analysis website (Zhou et al., 2019), Metascape was used to perform the enrichment analysis of hypermethylated and hypomethylated genes using the following ontology sources: Gene Ontology (GO) Biological Processes and Kyoto Encyclopedia of Genes and Genomes (KEGG) Pathway. Terms with a minimum overlap of 3, *p*-value < 0.01, and enrichment factor >1.5 were considered. Next, we used the STRING database to perform protein-protein interaction (PPI) network analysis of differentially methylated genes and the Molecular Complex Detection (MCODE) plug-in to analyze closely connected network components. Differentially expressed genes (DEGs; | FC| > 1.5, *p* < 0.01) methylated by m^5^C were used to further analyze the effect of m^5^C methylation. GO functional analysis was performed using the GO website, and terms with *p*-values < 0.05 were considered statistically significant. Moreover, KEGG pathway analysis was performed by DAVID (Huang da et al., 2009), and pathways with a *p*-value < 0.05 were considered to be significant. Gene set enrichment analysis (GSEA) was analyzed by the biological process items, and the mouse gene set data were downloaded from GSKB^1^.

### Methylated RNA Immunoprecipitation and Quantitative Reverse-Transcription PCR

Methylated RNA immunoprecipitation (MeRIP) was performed by Cloudseq Biotech, Inc. (Shanghai, China). Briefly, RNA was fragmented by incubation with fragmentation buffer at 94°C for 4 min. Stop solution (0.05 M EDTA) was added to stop fragmentation. The anti-m^5^C antibody (Diagenode, Cat# C15200081-100) and protein G beads were incubated with the fragmented RNA in IP buffer for 2 h on a rotating wheel at 4°C to reduce nonspecific binding. After the beads settled to the bottom of the tube, the supernatant was discarded, and IP buffer was added to resuspend the magnetic beads. This procedure was repeated twice, and the tube was placed on ice. The eluent was added and incubated at 4°C for 1 h, and clear supernatant was collected and purified with an RNase MiniElute Kit. The eluted sample was used to obtain RNA after MeRIP. RNA after MeRIP was analyzed by Quantitative Reverse-Transcription PCR (qRT-PCR) together with the corresponding input RNA. The primers used for qRT-PCR analysis are presented in Table 1.

**TABLE 1:** MeRIP qRT-PCR primer information.

**Name**	**Primer sequence (5′-3′)**	
Ngp	Forward: AAGGGGCCAAGAGTGGTAGT	Reverse: TAGTTGTCGAAGGGCCTCAC
Hbb-bs	Forward: TGCACCTGACTGATGCTGAG	Reverse: ACTTCATCGGCGTTCACCTT
Anxa1	Forward: GAGTCTCTCTTCAGTCCCCG	Reverse: GAAAACGGGCCTGCTTGAGG
Glrx5	Forward: GCTCTGTAAGCCCTGGAGTG	Reverse: CCACCTTGTCCTTCTTCACCA
Dpp4	Forward: AGAGAAGAGGGAGCAGGGAG	Reverse: AGTCTGGCAGTGAACAGCTC
Myo7a	Forward: GGCTCGGAGGAAGAAGGAAC	Reverse: TCCCCAGGAAGCCAAACATC
Prr13	Forward: GTGCGAACCCAGACTGAGAA	Reverse: GGAGGCCTTTAAGCATCCGT
Cyba	Forward: AGTGAGGACTTGCGAAGTGG	Reverse: TGTGTGAAACGTCCAGCAGT
Arhgap12	Forward: TGGCCGAGAGAAGTGGAAAG	Reverse: CAGGTCTGACTTGCCACCAG
Ywhah	Forward: ATGGGGGATCGAGAGCAG	Reverse: GGAGGCCATATCGTCGTAGC
Acta2	Forward: AAGAGGAAGACAGCACAGCC	Reverse: GGAGCATCATCACCAGCGAA
Heph	Forward: TTTGCCCTACCAGCTCAGTG	Reverse: TACACACTTCCTTTGCCCCG

### Immunocytochemistry

The cultured neurons were fixed with 4% paraformaldehyde for 10 min at room temperature. Then, 0.2% Triton X-100 was used to penetrate the membrane of the neurons, and the neurons were incubated with 10% goat serum (Solarbio, China) for 30 min. Next, neurons were incubated with primary antibodies overnight at 4°C [anti-β-III tubulin (1:400, Abcam Cat# ab78078) and anti-GFAP (1:400, Abcam Cat# ab7260)]. The next day, the neurons were incubated with the secondary antibody for 1 h at room temperature. Finally, cells were incubated with DAPI (Beyotime Biotechnology, China) for 10 min, and images were taken on a fluorescence microscope (Olympus, Japan).

### Western Blot

After the cells were washed with PBS to remove the influence of other protein substances in the medium, RIPA lysate (Solarbio, China) containing protease inhibitors was added to completely lyse the cells at 4°C for 30 min followed by centrifugation at 13,000 × *g* at 4°C for 10 min. The supernatant was collected, and the PierceTM BCA Protein Assay Kit (Thermo Scientific) was used to determine the protein concentration. Finally, the proteins were heated in a metal heater at 100°C for 10 min for denaturation. 25 micrograms of protein were separated by 12% SDS-PAGE and transferred to PVDF membranes. Membranes were blocked with 5% skim milk at room temperature for 1 h and then incubated with primary antibodies on a shaker at 4°C overnight using the following antibodies: Anti-β-Actin (1:10000; MBL International, Cat# JM-3598R-100, RRID:AB_2784536), Anti-Caspase-3 (1:1000; Cell Signaling Technology, Cat# 9662, RRID:AB_331439), Anti-Bcl-2 (1:1000; Cell Signaling Technology, Cat# 2870, RRID:AB_2290370), and Anti-Bax (1:1000; Cell Signaling Technology, Cat# 2772, RRID:AB_10695870). After washing in TBST solution contained 0.1% Tween, the immunoblot bands were visualized by using horseradish peroxidase-linked anti-rabbit IgG (1:3000; Cell Signaling Technology, Cat# 7074S, RRID:AB_2099233), and ImageJ software was used for grayscale value analysis.

### Statistical Analysis

Statistical analysis related to bioinformatics were performed using R software package (unless the method described otherwise) for statistical calculation. The experimental data was analyzed by unpaired *t*-test using GraphPad Prism 8 software, the data were expressed as mean ± standard deviation (SD) and *P* < 0.05 was considered statistically significant.

## Results

### mRNA m^5^C Profiling of Neurons and Verification of Methylation Positions

**FIGURE 1:**
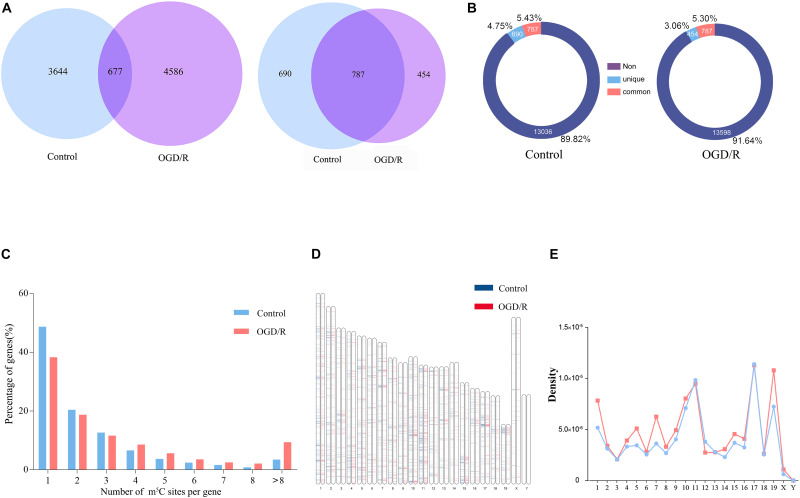
Overview of the mRNA m^5^C profiles in the control group and OGD/R group and verification of methylation targeting. **(A)** The overlap of the mRNA m^5^C sites (left) and genes methylated with the m^5^C site (right) between the control group and the OGD/R group. **(B)** Analysis of the proportion of m^5^C-modified genes. Non, genes with no methylation site; unique, the methylation sites only exist in the genes of OGD/R group or the control group; common, the methylation sites exist in the genes of both groups **(C)** Bar chart presenting the numbers of mRNA m^5^C sites per gene. **(D)** Visualization of the m^5^C sites at the chromosome level in the control group and OGD/R group. **(E)** Line graph presenting the density of m^5^C sites in each chromosome.

Bisulfite sequencing of neuronal RNAs was performed to obtain an overview of the transcriptome-wide m^5^C profiles (Supplementary Table 1). In general, we discovered 4321 methylation sites in the control group and 5263 methylation sites in the OGD/R group, and most recognition sites were specific in the two groups, corresponding to 84.33% of the control group and 87.14% of the OGD/R group (Figure 1A and Supplementary Figure 1A). Mapping of methylation sites to the mouse mm10 genome showed that the m^5^C sites were identified in neurons located in 1477 (control) and 1241 (OGD/R) annotated genes (Figure 1A), and the methylated genes accounted for 10.18% (control) and 8.36% (OGD/R) of genes with expression identified in neurons, respectively, (Figure 1B). Interestingly, we found that 48.6% of total methylated mRNAs had one m^5^C site in the control group, whereas this proportion was reduced to 38.3% in the OGD/R group (*p* = 7.36e-05). In addition, mRNAs with more than eight m^5^C sites accounted for 3.4% (control) and 9.3% (OGD/R) of total mRNAs, respectively, (*p* = 3.36e-05). Compared with that in the control group, the number of m^5^C sites per mRNA in the OGD/R group increased significantly (Figure 1C). The graphics of the methylation sites on the chromosomes were visualized using the R software package according to the reported data and code, indicating that the distribution of m^5^C sites on each chromosome was different between the two groups, especially on chromosomes one, five, seven and nineteen (Figures 1D,E). Moreover, few m^5^C sites were located on sex chromosome X, and no m^5^C sites were noted on chromosome Y in both groups, which seems to be related to the incidence of tissue specificity (Figures 1D,E).

**FIGURE 2:**
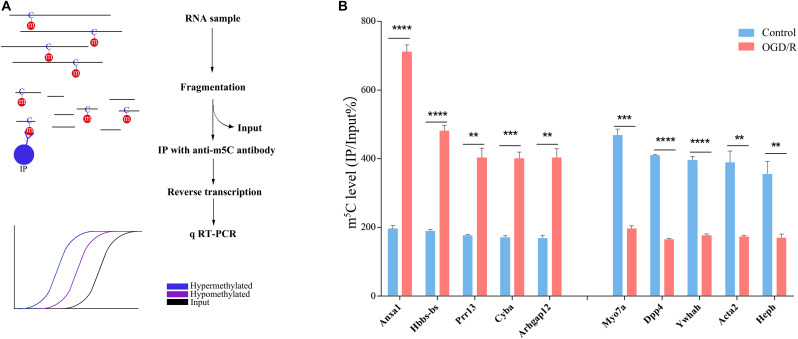
verification of m^5^C methylation positions. **(A)** Illustration of the MeRIP approach. RNA was extracted from neurons, chemically lysed and then divided into the IP group and input group. The IP group was incubated with anti-5-methylcytosine antibody. Finally, the expression level of candidate transcripts was analyzed by qRT-PCR. The blue line represents the hypermethylated transcripts, the purple line represents the hypomethylated transcripts, and the black line indicates the enrichment of the input group. **(B)** m^5^C level changes in 5 candidate hypermethylated transcripts and 5 candidate hypomethylated transcripts in the control and OGD/R groups (fold change > 2, *p* < 0.01). **p < 0.01; ***p < 0.001; ****p < 0.0001.

To verify the accuracy of the RNA-BS-seq results, we selected 10 candidate transcripts divided into hypermethylated group and hypomethylated group according to the sequencing data (Supplementary Figure 2), and the m^5^C methylated state was confirmed by methylated RNA immunoprecipitation (MeRIP; Figure 2A). Among the 10 candidate transcripts analyzed, 5 transcripts were hypermethylated (Anxal, Hbbs-bs, Prr15, Cyba, and Arbgap12), and 5 transcripts were hypomethylated after OGD/R (Myo7a, Dpp4, Ywhah, Acta2, and Heph). The MeRIP verification results are completely consistent with the sequencing results; thus, we are confident that our data represent a reliable image of m^5^C modification of neuronal transcripts (Figure 2B).

### Common and Distinct Distribution Features of mRNA m^5^C Sites Identified in Neurons Before and After OGD/R Treatment

**FIGURE 3:**
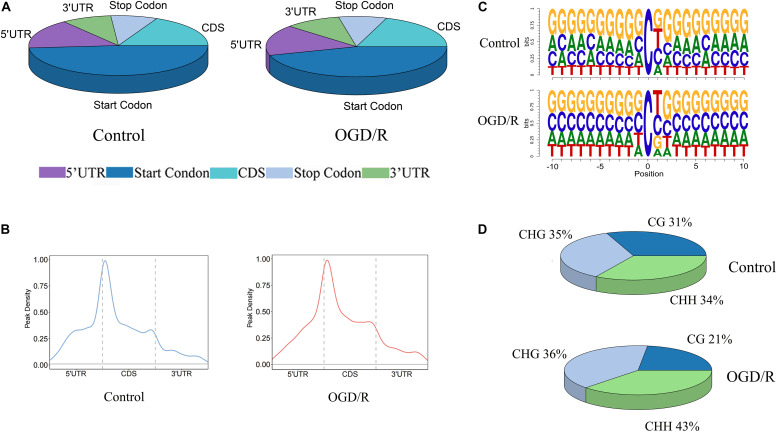
The distribution patterns of m^5^C sites of mRNA. **(A)** Pie chart of the distribution of m^5^C sites in the indicated regions **(B)** The peak density of mRNA m^5^C sites distributed along mRNA transcripts. The dashed lines represent the translational start and stop codons. **(C)** Sequence frequency logo of the sequences close to the mRNA m^5^C sites of the two groups. **(D)** Proportions of mRNA m^5^C sites in each sequence context: CG, CHG and CHH, where H = A, C, or U.

To further understand the distribution of m^5^C methylation on mRNA, we separately analyzed the distribution of m^5^C sites in the control group and OGD/R group. The positions of m^5^C were divided into five different regions according to their locations in transcripts. The descending order of the degree of m^5^C modification was start codon, coding sequence (CDS), 5′-untranslated region, stop codon and 3′untranslated region (Figure 3A). It’s worth noting that the distribution patterns of m^5^C sites in the control and OGD/R groups were similar. In addition, further analyses indicated that the m^5^C sites were mainly enriched in regions immediately downstream of translation initiation sites in both groups (Figure 3B). Similarly, a sequence frequency logo showed that m^5^C methylation was embedded in CG-rich environments regardless of whether it had undergone OGD/R treatment (Figure 3C). Moreover, the methylation sites are distributed in three types of special sequence contexts (CG, CHG and CHH, where H = A, C, or U). After OGD/R treatment, the number of CG regions significantly decreased from 31 to 21%, while the number of CHH regions markedly increased from 34 to 43% (Figure 3D).

**FIGURE 4:**
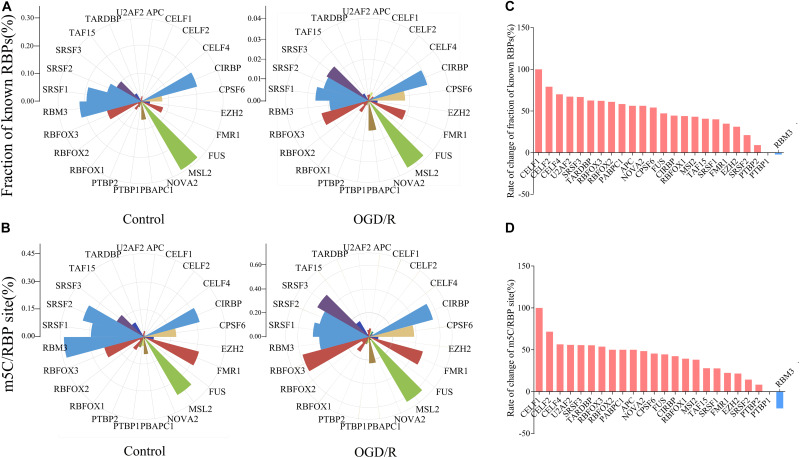
Rose diagrams depict an overlap of mRNA m^5^C sites with RBP-binding sites available in POSTAR. **(A)** Fraction of binding sites overlapping with a m^5^C site for each particular RBP. Fraction of known RBPs = RBP binding sites overlapping with a m^5^C site/RBP binding sites. **(B)** The number of m^5^C sites overlapping with a binding site of a particular protein was normalized against the total number of m^5^C sites. m5C/RBP site = m^5^C sites overlapping with a RBP binding site/RBP binding sites. **(C,D)** Histograms present the difference between two groups. The result is the difference between the number of binding sites in the OGD/R group and the control group divided by the number of sites in the OGD/R group. (**C**, Fraction of known RBPs; **D**, m^5^C/RBP).

We next jointly analyzed the mRNA m^5^C site profile and the binding sites of RBPs in the POSTAR database to determine the relationship between the methylation sites and protein-binding sites (Supplementary Table 2). According to the transcriptome sequencing data, 24 RBPs with higher gene expression level were selected, and approximately 18.21% of m^5^C sites in the control group and 22.11% of OGD/R group m^5^C sites overlapped with the mapped RBP binding sites. In particular, RBFOX1, RBFOX2, RBFOX3, APC, CELF4, TARDBP, U2AF2, FUS, and FMR1 correlated with brain tissue (Figures 4A,B). After OGD/R treatment, the overlap probability of m^5^C sites with the binding sites of most RBPs increased, except for the RBM3 binding protein (Figures 4C,D). The analysis showed that OGD/R treatment obviously changed the overlapping probability of RBP-binding sites with m^5^C sites, indicating that m^5^C methylation may indirectly regulate gene function by interacting with RBPs.

### Functional Analysis of Differentially Methylated m^5^C-Modified Genes Between the Control and OGD/R Groups

**FIGURE 5:**
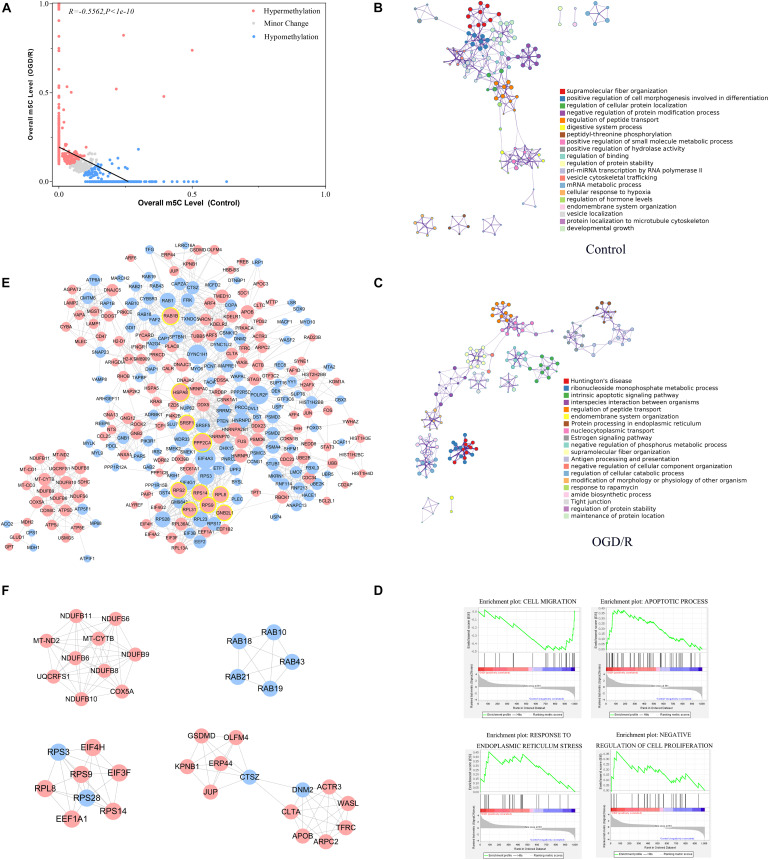
Functional analysis of differentially methylated m^5^C-modified genes between the control and OGD/R groups. **(A)** Relationship of the methylation level of m^5^C transcripts between the control and OGD/R groups. Red dots represent transcripts with increased levels of m^5^C modifications after OGD/R, whereas blue dots represent opposite attributes. Regression lines are shown, and the Pearson correlation coefficient (R) was calculated by GraphPad Prism. **(B,C)** Network of enriched terms colored by cluster ID for differentially methylated genes (**B**, control group; **C**, OGD/R group). Each term is represented by a circular node, where its size is proportional to the number of input genes that are related to that term and its color represents its cluster identity. Terms with a similarity score > 0.3 are linked by a line (thickness of the line represents the similarity score). **(D)** GSEA of differentially m^5^C-modified genes with GO gene sets. **(E)** PPI network analysis of differentially expressed m^5^C-modified genes. Red nodes represent hypermethylated genes, and blue nodes represent hypomethylated genes. The top 10 nodes of hypermethylated genes are marked with yellow circle. **(F)** The 5 most meaningful modules selected from the PPI network using MCODE plug-in.

Previous studies have noted that after cerebral IRI, the m^6^A RNA demethylase Alkbh5 can selectively prevent the degradation of the Bcl-2 protein, which may play an important role in reducing nerve apoptosis and protecting nerve function (Xu K et al., 2020). The m^5^C site has been confirmed to be abundant in brain tissue (Amort et al., 2017), whereas the role of the m^5^C RNA modification in cerebral IRI remains unclear. By analyzing the methylation level of methylated transcripts, we strictly filtered the methylation site information (Supplementary Table 3) and identified 480 hypermethylated transcripts and 382 hypomethylated transcripts in neurons after OGD/R (Figure 5A and Supplementary Table 4). To understand the function of differentially methylated genes, we conducted Metascape analyses and found that the hypermethylated genes were mainly enriched in pathological processes, such as Huntington’s disease and intrinsic apoptotic signaling pathways, whereas hypomethylated genes were mainly enriched in physiological processes (Figures 5B,C). To further confirm the biological function of neurons between the control and OGD/R groups, we performed GSEA and focused on important biological functions, including response to endoplasmic reticulum stress, apoptotic process, negative regulation of cell proliferation, and cell migration (Figure 5D). The results indicated that m^5^C modification was more abundant in cellular stress and cell death-related gene sets after OGD/R treatment, which seemed to be closely related to cerebral injury.

In addition, the PPI network showed that HSPA8, PPP2CA, RPS9, RPL8, RPS14, RAB1B, RPS2, SRSF1, GNB2L1, and RPL31 were the top 10 high−degree nodes of hypermethylated genes, which may play important roles in the pathological processes of the brain (Figure 5E). Heat shock protein family A (Hsp70) member 8 (HSPA8) played an important role in the occurrence and development of neurological diseases, such as Alzheimer’s disease and Parkinson’s disease (Lauterbach, 2013; Silva et al., 2014). Ribosomal protein L8 (RPL8) expression levels were significantly increased in the brain tissue after cerebral hemorrhage, and Ribosomal protein S9 (RPS9)expression was increased significantly in the neurons of the dentate gyrus after acute cerebral ischemia (Kim et al., 2003; Chen et al., 2019a). By analyzing the functions of the main gene modules through MCODE, we found that the main methylation gene modules were significantly related to basic life activities of cells, including gene expression, biosynthetic processes, energy metabolism and transport (Figure 5F), and these results were similar to previous studies (Yang et al., 2019).

### Integration Analyses of m^5^C-Containing mRNA Methylation and mRNA Transcript Expression

The principal component analysis (PCA) results of transcripts indicated significant differences between the gene expression patterns of the two groups, whereas the expression patterns were similar within the same group (Supplementary Figure 3A). GO analysis showed that up-regulated DEGs after OGD/R were enriched in specific biological processes (e.g., sensory perception of chemical stimulus, cellular response to stress, and regulation of cell death), molecular functions (e.g., transmembrane signaling receptor activity), and cellular components (e.g., nucleus, non-membrane-bounded organelle, and organelle lumen). However, down-regulated DEGs were enriched in the items closely related to essential neuronal processes and components, including neurogenesis, neuron projection development, generation of neurons, neuron differentiations, axon, and dendritic tree (Supplementary Figure 3B). In addition, KEGG pathway analysis indicated that down-regulated DEGs were generally linked to basic neuronal development pathways, whereas up-regulated DEGs were interestingly enriched in some cancer-related pathways (Supplementary Figure 3C).

**FIGURE 6:**
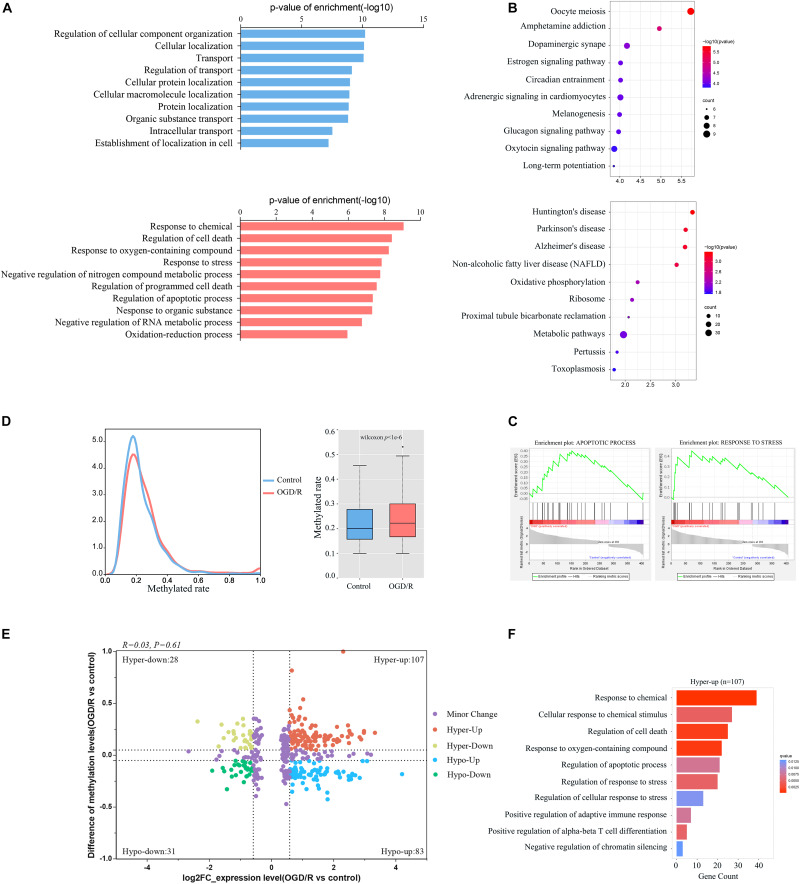
Integration analyses of m^5^C-containing mRNA methylation and mRNA transcript expression. **(A)** GO biological process analyses of DEGs modified by m^5^C in control and OGD/R neurons. **(B)** KEGG analysis of DEGs modified by m^5^C in the control group and OGD/R group. **(C)** GSEA of DEGs modified by m^5^C with Gene Ontology sets. **(D)** Comparison of m^5^C levels in mRNAs between the control group and OGD/R group, density distribution plot (left), and box plot (right). **(E)** mRNA distributions with significant changes in gene expression and m^5^C modification levels between the control group and OGD/R group. **(F)** GO biological process analysis of hypermethylated mRNAs with upregulated genes after OGD/R treatment.

Next, we integrated RNA-Seq data and RNA-BS-seq data to co-analyze the m^5^C-methylated DEGs for the further exploration of the function of m^5^C modification (Supplementary Table 5). GO terms of the control group were mainly enriched in physiological processes, such as substance transport, cell location and adhesion signaling, whereas GO terms in the OGD/R group were mainly enriched in the cell stress response and cell death processes (Figure 6A). KEGG analysis also showed that genes upregulated after OGD/R were mainly enriched in neurological disease pathways, such as Huntington’s disease, Parkinson’s disease and Alzheimer’s disease, whereas downregulated DEGs were mainly enriched in normal physiological pathways (Figure 6B). Finally, the GSEA results indicated that gene sets of apoptotic processes and response to stress were significantly up-regulated (Figure 6C). Moreover, our analysis revealed that the level of methylation increased significantly after OGD/R (Figure 6D), while the four-quadrant diagram indicated that a strong correlation did not exist between the mRNA m^5^C modification level and the expression level (Figure 6E), and similar results were previously reported in systemic lupus erythematosus (SLE; Guo et al., 2020). We further investigated the biological process enrichment of mRNAs that were both upregulated and hypermethylated using GO functional analysis. Particularly, these transcripts were enriched in the following categories: response to chemicals, cellular response to chemical stimuli, regulation of cell death and regulation of apoptotic processes (Figure 6F), which indicates that both hypermethylated and upregulated transcripts play an important role in neuronal damage caused by ischemia and hypoxia.

### Neuronal Apoptosis Occurs After OGD/R Treatment *in vitro*

To clarify the apoptotic status of neuron after OGD/R treatment, we extracted primary neurons from mice and successfully constructed a neuronal OGD/R model (Supplementary Figure 4A). A TUNEL assay was performed to examine apoptosis and the associated protein expression levels between the control and OGD/R models. TUNEL-positive cells detected in the OGD/R group exhibited increased DNA fragmentation, indicating that the neurons exhibited obvious apoptosis *in vitro* after OGD/R (Supplementary Figures 4B,C). In addition, western blot assay revealed that the protein expression levels of Cleaved-Caspase3 and Bax significantly increased after the OGD/R treatment, while the level of Bcl-2 decreased. The experimental results further confirmed that the neuronal damage caused by OGD/R was closely related to neuronal apoptosis (Supplementary Figures 4D,E).

## Discussion

An increasing number of studies have demonstrated that mRNA methylation is involved in many neural functions and has an important impact on life activities. The absence of Methyltransferase-like 14 (METTL14) in the central nervous system (CNS) can prolong the cell cycle of cortical progenitor cells and reduce the differentiation of radial glial cells (Yoon et al., 2017). YTH domain family 2 (YTHDF2) can regulate neurodevelopment by degrading m^6^A methylation levels of neuronal mRNA (Li et al., 2018). In addition, mRNA methylation also plays an important role in the maturation of oligodendrocytes and the myelination of the CNS (Xu H et al., 2020). To date, most research on mRNA methylation in the nervous system has focused on m^6^A modification. As a new type of RNA methylation, m^5^C has been proven to play important roles in promoting RNA export out of the nucleus, regulating protein translation, and neural stem cell differentiation (Schaefer et al., 2009; Li et al., 2017). Studies have shown that m^5^C methylation is enriched in mouse brain tissues (Amort et al., 2017), whereas the specific distribution and function of mRNA m^5^C in neurons and its role in the important pathological processes of IRI remain unclear.

In this study, we conducted a comparative analysis of cytosine methylation in neuronal mRNA before and after OGD/R treatment, revealing the m^5^C modification of mRNA in neurons for the first time. We also clarified the main differences in neuronal m^5^C modification after OGD/R, including the methylation sequence preference, proportion of neuronal methylated mRNA, mRNA methylation modification level, distribution characteristics of methylated sites in mRNA and the distribution of methylated transcripts in chromosomes. Notably, the total methylation level in a sample depends on the modification level of each transcript and the number of methylated transcripts. In our results, we observed that after OGD/R treatment, the number of methylated sites increased significantly, but the number of methylated transcripts decreased. Compared with that of the control group, the transcript modification level of the OGD/R treatment group was increased, and there were more mRNAs with abundant methylation sites. Motif analysis shows that C sites were enriched in GC-rich regions, which is consistent with the results of previous studies (Yang et al., 2017).

Cytosine methylation accumulates near the translation initiation codon, which is similar to the previously reported distribution pattern of m^5^C in mouse brain tissues. In contrast, neuronal mRNA m^5^C sites are not significantly enriched in the 3′UTR region and are relatively more concentrated near the start codon and adjacent 5′UTR and CDS regions. Studies have demonstrated that the distribution patterns of m^5^C in mice and humans are highly conserved, and these sites are concentrated near the start codon, which is similar to the results noted for m^6^A modifications (Dominissini et al., 2012). Moreover, our results show that the distribution patterns of m^5^C in neuronal mRNA before and after OGD/R treatment are very close, proving that the distribution of m^5^C on mRNA remains stable under different conditions. However, m^5^C in *Arabidopsis* is mainly concentrated in the CDS area (Cui et al., 2017), exhibiting a different distribution pattern and indicating that the m^5^C modification of mRNA differs between animals and plants. This may be related to regulatory effects of m^5^C methyltransferases and bind proteins. At present, NSUN2 has been found to be the methyltransferases involved in the m^5^C modification of mammalian mRNA, which plays an important role in transcript translation (Tang et al., 2015). In addition, m^5^C methylation-binding nucleoprotein ALYREF can promote mammalian mRNA export and YBX1 promotes mammalian mRNA stability (Yang et al., 2017; Chen et al., 2019b). The main methylase found in plants is TRM4B, which plays an important role in the stability of tRNA and has a negative regulatory effect on mRNA expression (David et al., 2017). Moreover, previous studies discovered that cytosine methylation of mRNA in eukaryotes can effectively promote mRNA translation (Delatte et al., 2016), which may also be related to the distribution of m^5^C enriched around the start codon. RBPs play an important role in the regulation of mRNA expression. Furthermore, studies have shown that suppression of RBP SRSF1 prevents neurodegeneration and motor dysfunction in C9ORF72-related diseases (Hautbergue et al., 2017). After hypoxic-ischemic brain injury, RBM3 promotes neurogenesis in a niche-dependent manner through the IMP2-IGF2 signaling pathway (Zhu et al., 2019). Our study indicated that the overlap sites of SRSF1 and RBM3 with m^5^C are significantly altered before and after OGD/R, which may be related to the pathological processes of hypoxia-reperfusion.

Previous studies have shown that m^5^C-modified mRNA is involved in various biological processes (Flores et al., 2017; Yang et al., 2019; Chen et al., 2019b). In this study, we compared neuronal m^5^C methylation information before and after OGD/R. The results showed that compared with the control group, the OGD/R group had 862 transcripts with different levels of m^5^C methylation, including 480 hypermethylated and 382 hypomethylated transcripts (*p* < 0.01). These transcripts may be related to the pathogenesis of IRI. Previous studies have found that Tet methylcytosine dioxygenase 3 (TET3) has ischemic neuroprotection function by promoting the formation of DNA hydroxymethylation in the brain after IRI, which mainly occurs through biological pathways involved in oxidative stress and DNA repair (Morris-Blanco et al., 2020). Our research found that after OGD/R, the hypermethylated transcript functions were mainly enriched in pathological processes, including Huntington’s disease and intrinsic apoptotic signaling pathways, whereas the hypomethylated transcript functions were mainly enriched in physiological processes. Moreover, the hub genes RPS9 and RPL8 of PPI network analysis were significantly increased in the brain tissue after cerebral ischemia and hemorrhage (Kim et al., 2003; Chen et al., 2019a). These findings indicate that m^5^C modification may also be involved in the pathological processes of cerebral injury.

To further explore the role of mRNA m^5^C modification in neurons, we performed bioinformatics analysis of 406 differentially expressed m^5^C-methylated transcripts, including 282 hypermethylated and 124 hypomethylated transcripts (*p* < 0.05). Our analyses did not reveal a strong relationship between the m^5^C methylation level and the corresponding mRNA expression level, which is similar to the results of previous reports on bladder cancer and SLE (Chen et al., 2019b; Guo et al., 2020), and previous studies have shown that m^5^C methylation plays an important role in promoting the process of nuclear export (Yang et al., 2017). Therefore, the specific regulatory mechanism of m^5^C on mRNA deserves further exploration. Our research found that after OGD/R treatment, neurons are in a state of stress, their responses to chemical and oxygenated compounds are significantly enhanced, and neuronal apoptosis is simultaneously initiated. Methylation modification has been confirmed to play an important role in regulating neuronal death during neuronal oxidative stress (Chen et al., 2019c), but the role of the m^5^C modification in these processes remains unknown. Then, we focused on the up-regulated methylated transcripts and found that up-regulated transcripts with hypermethylated modification were significantly enriched in the processes of apoptosis and neurological diseases. However, these processes were not enriched in hypomethylated transcripts, indicating that m^5^C modification may play an important role in the pathological process of neuronal apoptosis and other neurological diseases. In addition, mRNA m^5^C modification was closely related to the neuronal fate after IRI. However, further functional studies are still needed to clarify the relationship between the neuronal mRNA m^5^C modification and expression level after IRI.

In summary, our study presented the first transcriptome-wide m^5^C methylation map before and after neuronal OGD/R injury and found that the m^5^C modifications are highly conserved in mRNA. Furthermore, our results provide a potential relationship between differential m^5^C mRNA modifications and neuronal damage induced by OGD/R. This new epigenetic modification may provide better insights into the pathogenesis of related neurological diseases and exogenous nerve damage. However, it is unclear how neuronal m^5^C expression can be specifically regulated; for example, the specific role of RNA methyltransferases NSUN2 and DNMT2 in neuronal ischemic injury remains unknown. More research is needed to discover its internal regulation mechanism. Targeting the m^5^C modification will become a promising strategy for the treatment of ischemia/reperfusion injury in the future.

## Data Availability Statement

The raw data has been made publicly available. GEO accession: GSE165256.

## Ethics Statement

The animal study was reviewed and approved by Experimental Animal Ethics Committee of Tianjin Medical University.

## Author Contributions

HJ, CZ, ZQ, and XL conducted experiments and data collection. HJ, YoL, YK, and WD analyzed and interpreted the m^5^C data. YiL, CW, WW, SS, and MH contributed to statistical analysis. HJ wrote the manuscript. SF and HZ provided the project funding and revised the manuscript. All authors have read and agreed to the published version of the manuscript.

## Conflict of Interest

The authors declare that the research was conducted in the absence of any commercial or financial relationships that could be construed as a potential conflict of interest.

## Footnotes

1 http://ge-lab.org/gskb/
