# Supplementary material for: Alteration of mRNA 5-Methylcytosine Modification in Neurons After OGD/R and Potential Roles in Cell Stress Response and Apoptosis
Source: Front Genet. 2021 Feb 3;12:633681. doi: 10.3389/fgene.2021.633681 (PMC7887326; doi:10.3389/fgene.2021.633681)
Supplement: Supplementary file 1 [file Data_Sheet_1.PDF]

Supplementary Figures

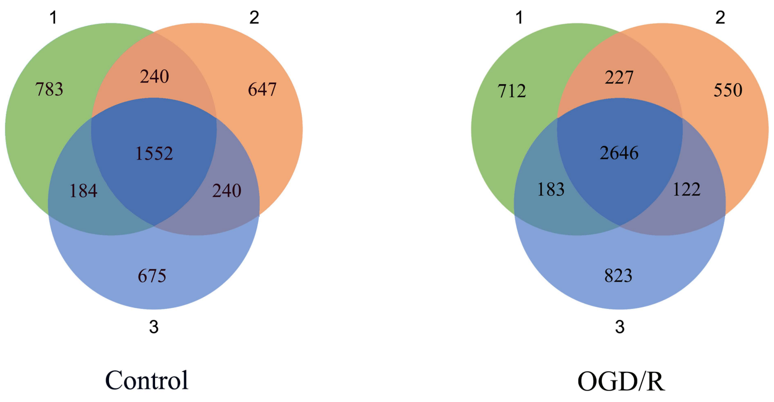

Supplementary Figure 1. Venn diagrams showing the overlap of mRNA m^{5}C sites between three sequencing pool replicates.

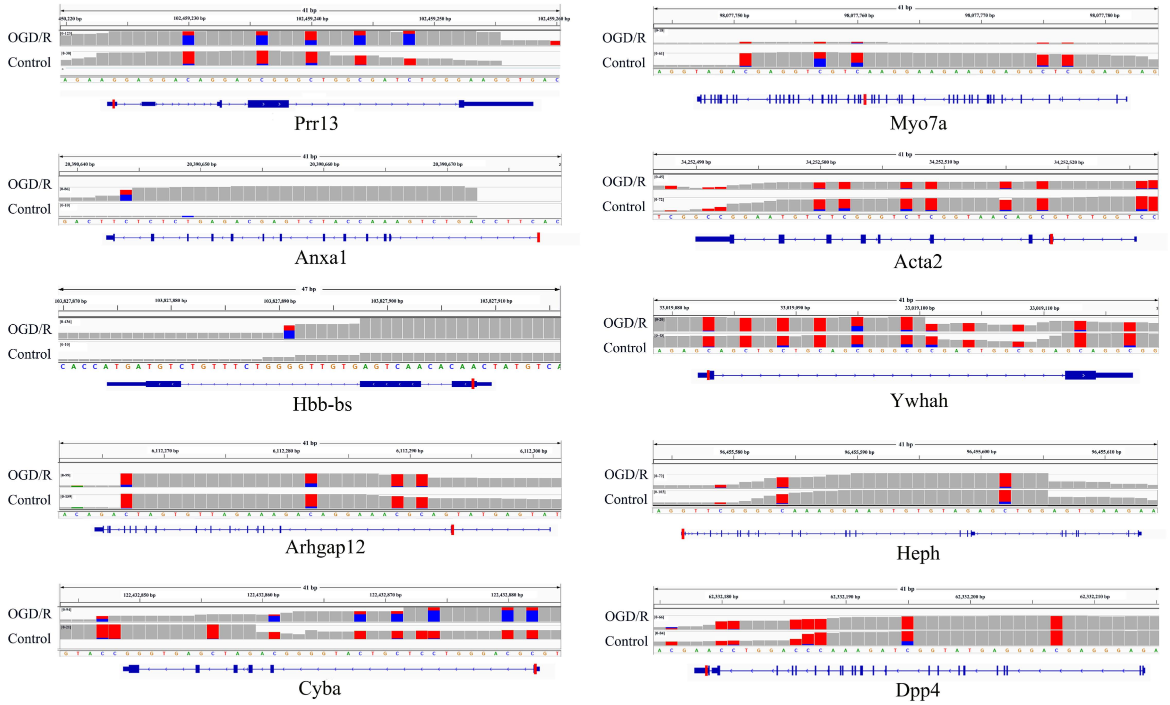

Supplementary Figure 2. According to RNA-BS-seq profiles, we randomly selected 5 hypermethylated fragments of different transcripts (left side) and 5 hypomethylated fragments of different transcripts (right side). Red indicates unmethylated C base (C base mutated to T base after the bisulfite treatment), and blue indicates methylated C base (no C base mutation occurred).

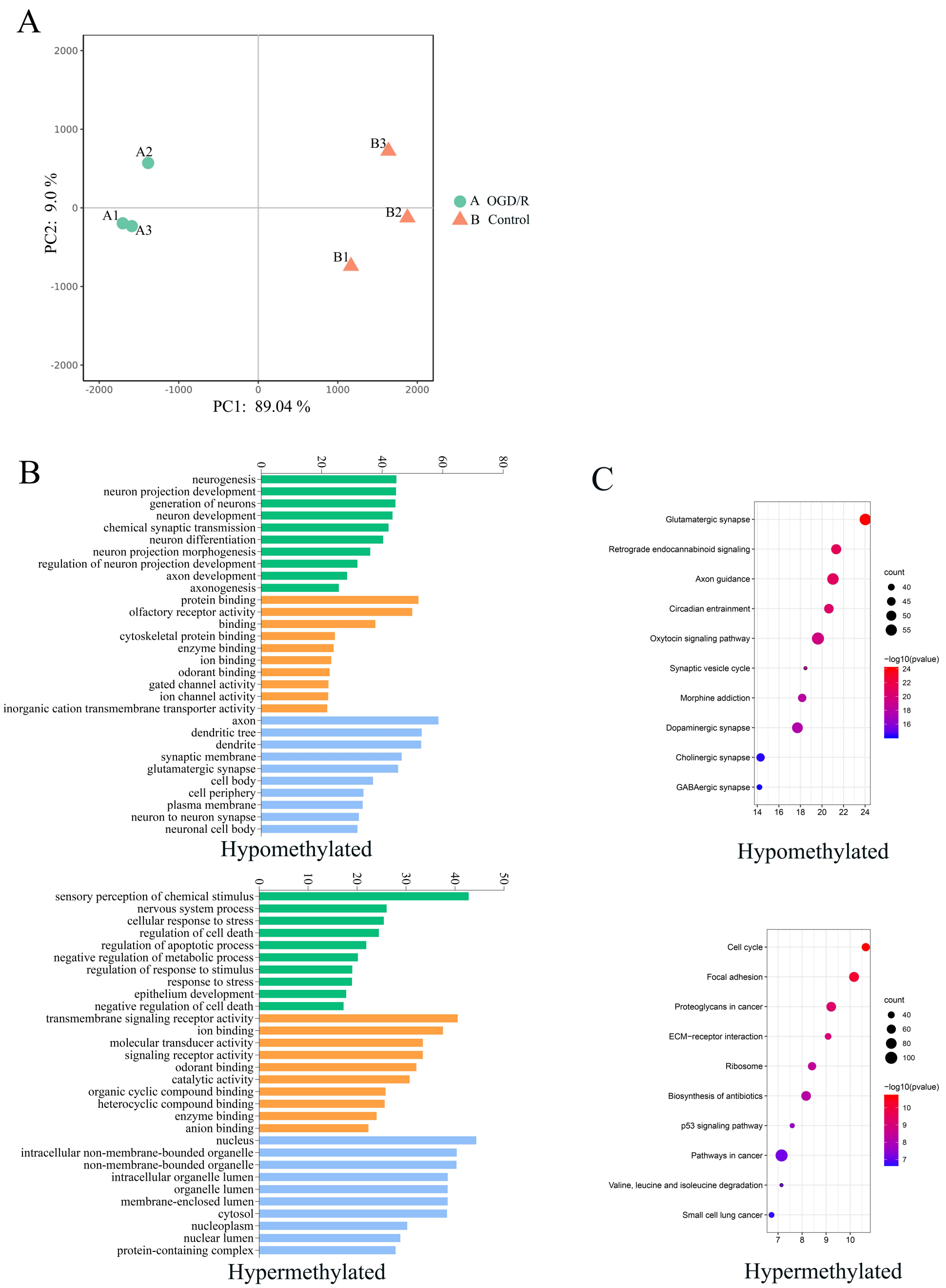

Supplementary Figure 3. Analysis of differentially expressed mRNAs after OGD/R treatment. (A) Principal component analysis (PCA) of the mRNA expression profiles in control and OGD/R neurons. (B) Gene Ontology analysis of differentially expressed mRNAs. Green indicates biological process (BP), orange indicates molecular function (MF), and blue indicates cellular component (CC). (C) KEGG pathway analysis of differentially expressed mRNAs.

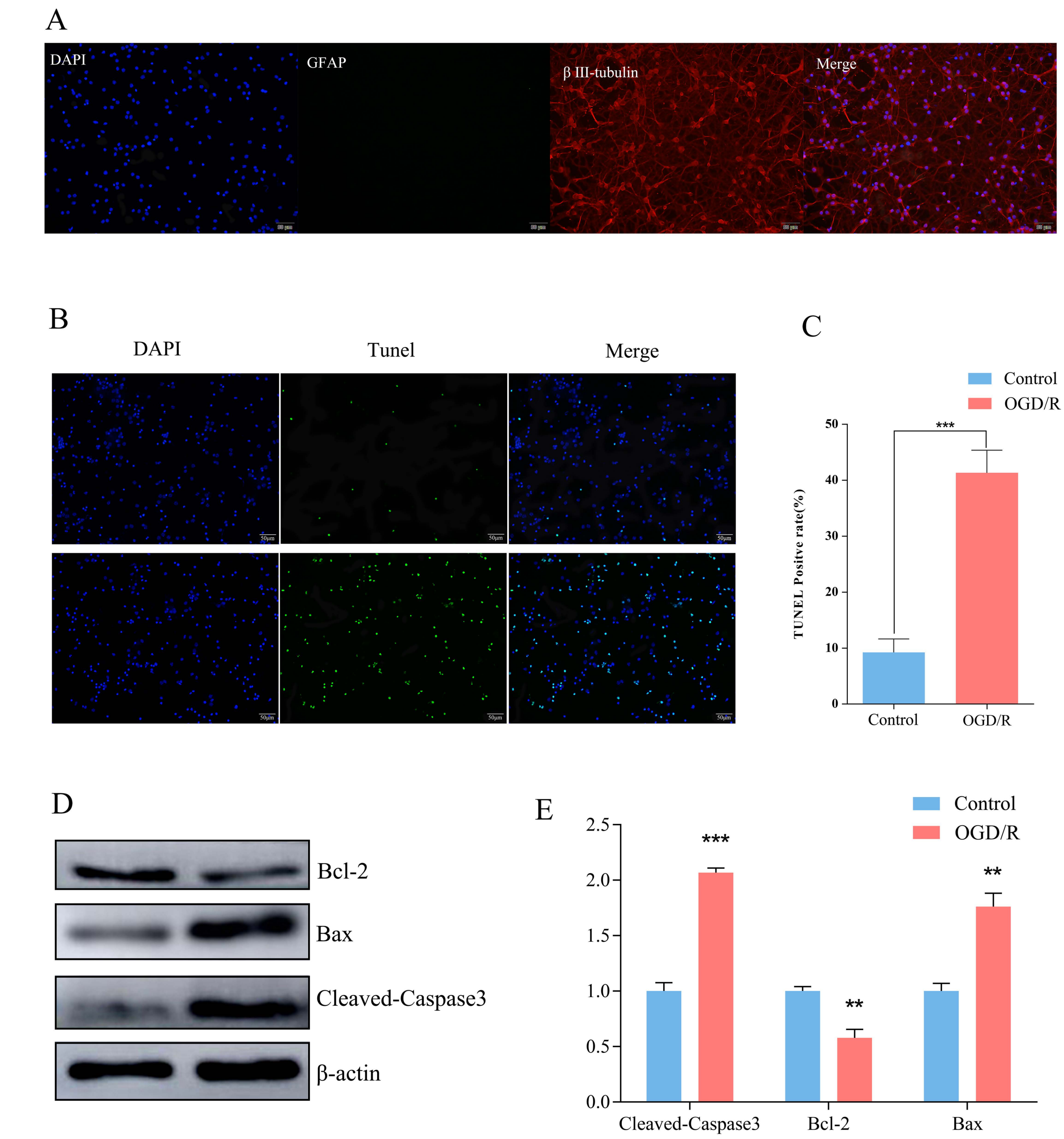

Supplementary Figure 4. Neuronal apoptosis occurs after OGD/R treatment in vitro. (A) Identification of the primary neurons after isolation for 6 days. Neurons were identified with an anti-β-III tubulin antibody and GFAP staining to determine the presence of astrocytes, and nuclei were stained with DAPI, Scale bars = 50 μm. (B) Neuronal apoptosis phenotype identified by a TUNEL assay: DAPI-stained nuclei are blue and TUNEL-positive cells are green. (C) The red column indicates the percentage of TUNEL-positive cells after OGD/R, which is shown with a blue column in the control group. (D) Identification of apoptotic protein markers by a western blot assay. (E) The grayscale was calculated by ImageJ and compared between the control group and the OGD/R group ($p < 0.01$).
